# Supplementary material for: ALKBH5-mediated m6A modification of circFOXP1 promotes gastric cancer progression by regulating SOX4 expression and sponging miR-338-3p
Source: Commun Biol. 2024 May 14;7:565. doi: 10.1038/s42003-024-06274-7 (PMC11094028; doi:10.1038/s42003-024-06274-7)
Supplement: Supplementary file 2 — Supplementary Information [file 42003_2024_6274_MOESM2_ESM.docx]

Representative uncropped western blot images.


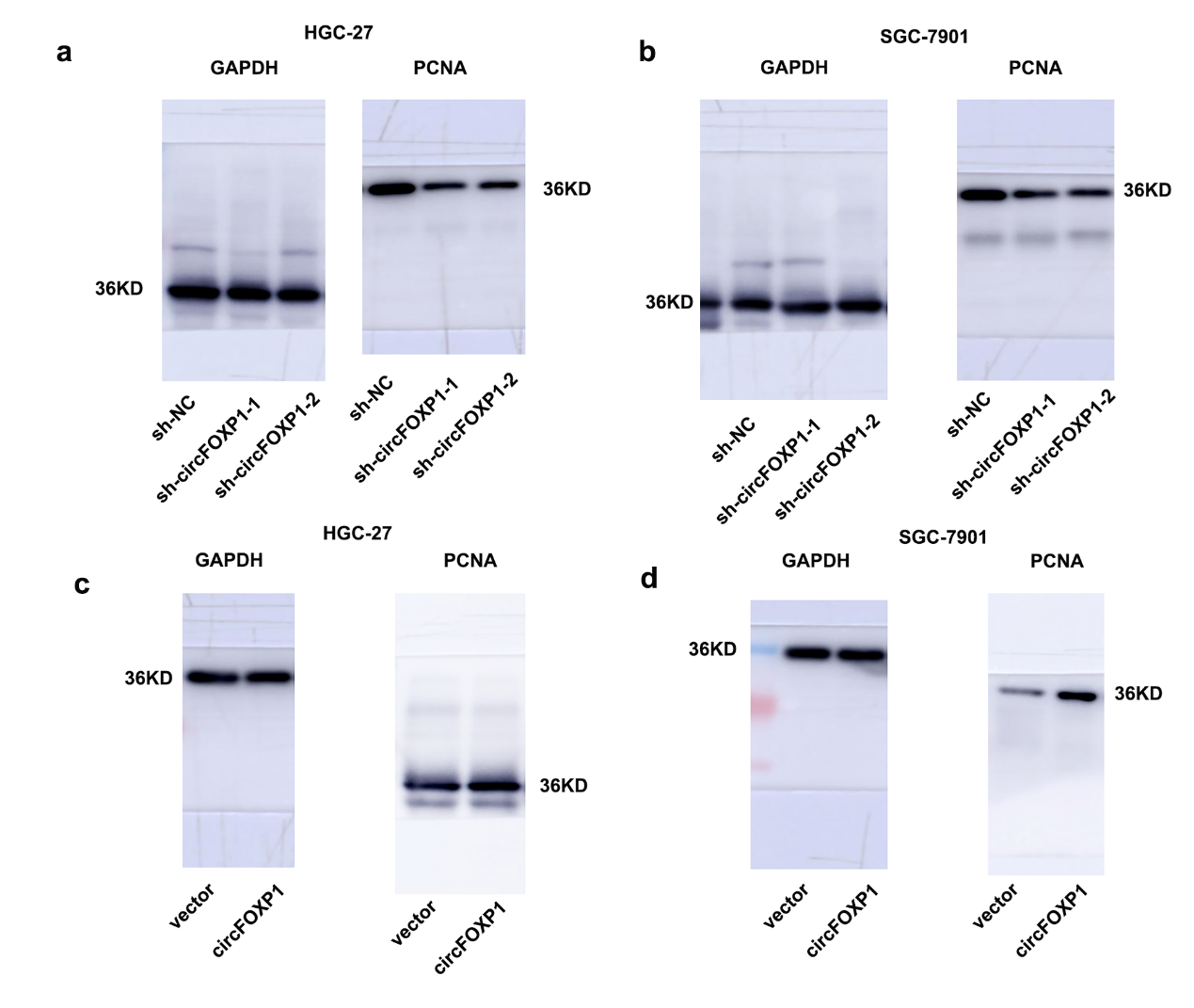


**Supplementary Fig. 1Western blotting results of PCNA expression (Fig. 2e) a, b** circFOXP1 knockdown remarkably inhibited PCNA expression in HGC-27 or SGC-7901 cells compared to the control group. **c, d** circFOXP1 overexpression remarkably enhanced PCNA expression in HGC-27 or SGC-7901 cells compared to the control group.


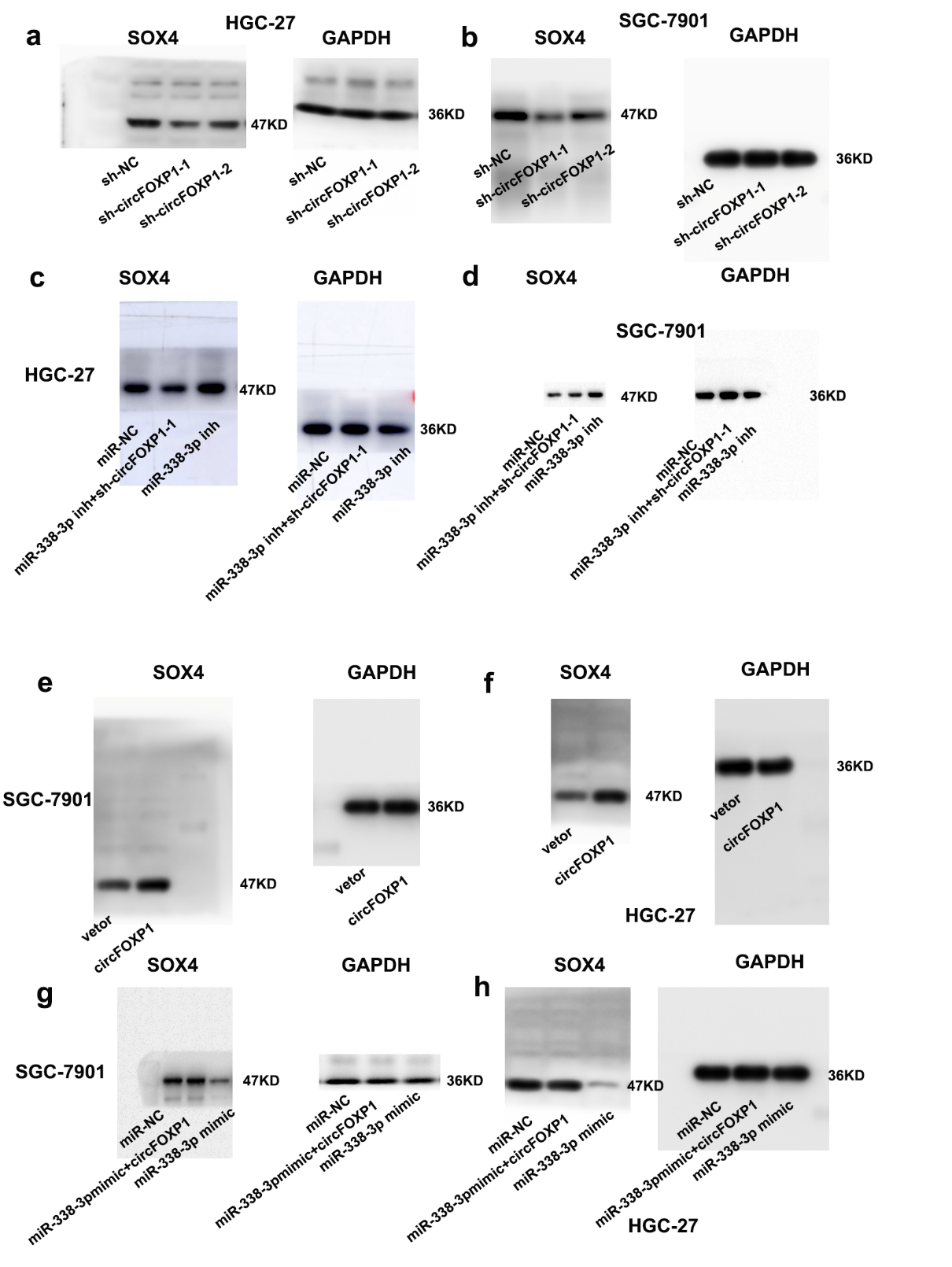


**Supplementary Fig. 2 Western blotting results of SOX4 expression (Fig. 4i, Fig 4j) a, b，c，d** The relative protein expression of SOX4 was detected after transfection of HGC-27 and SGC-7901 cells with sh-NC, sh-circFOXP1-1 or sh-circFOXP1-2 or miR-NC, miR-338-3p inhibitor or co-transfection with miR-338-3p inhibitor and sh-circFOXP1-1. **e, f, g, h** The relative protein expression of SOX4 was detected after transfection of HGC-27 and SGC-7901 cells with pLCDH-vector, pLCDH-circFOXP1 or miR-NC, miR-338-3p mimic, co-transfection with pLCDH-circFOXP1 and miR-338-3p mimic.


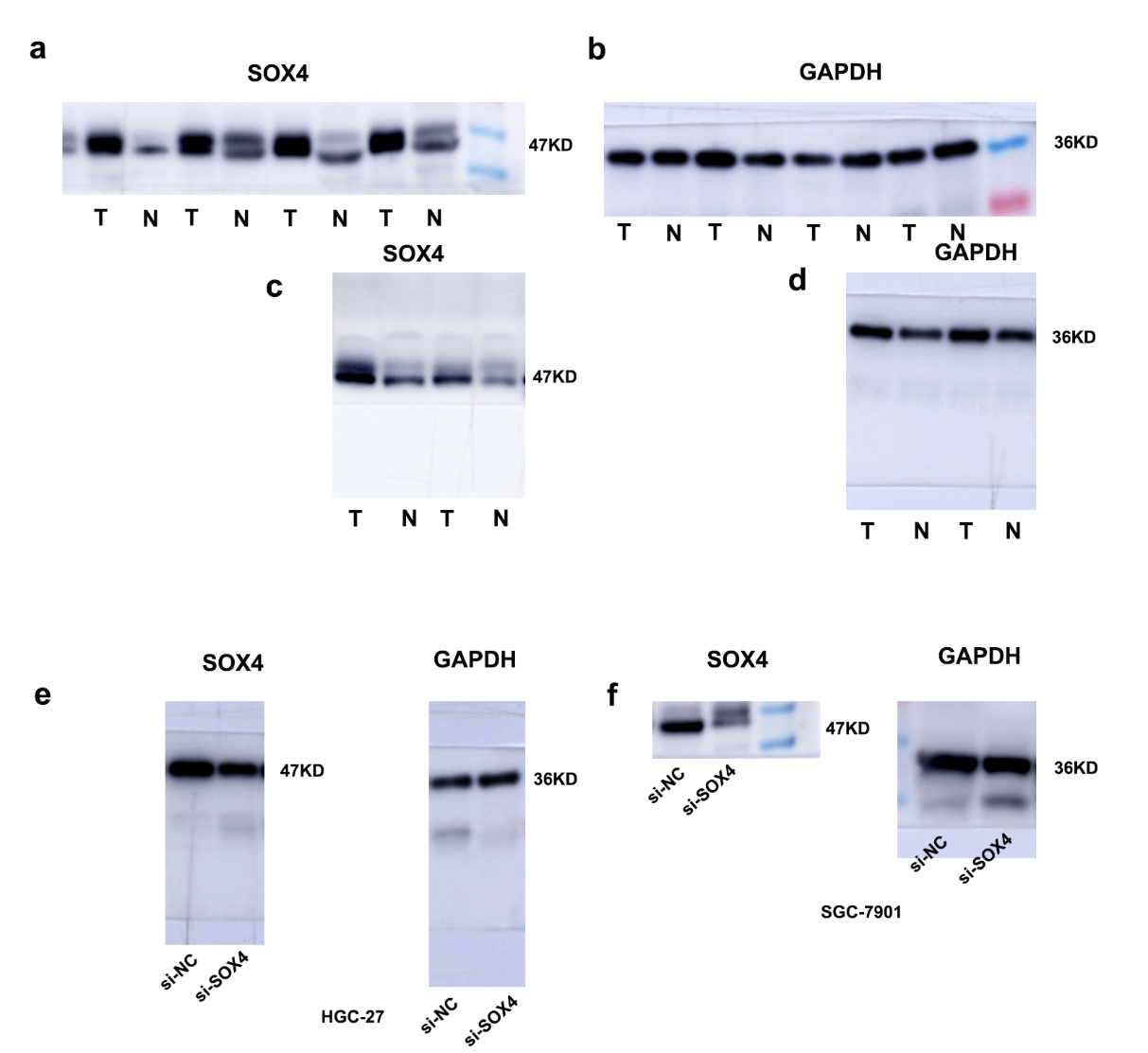


**Supplementary Fig. 3 Western blotting results of SOX4 expression (Fig. 5a, Fig 5b) a, b, c, d** SOX4 expression levels were evaluated by western blot analysis in human tissues from 56 cases of GC compared with adjacent normal tissues. **e, f** The relative protein expression of SOX4 was detected after transfection of HGC-27 and SGC-7901 cells with si-NC and si-SOX4.


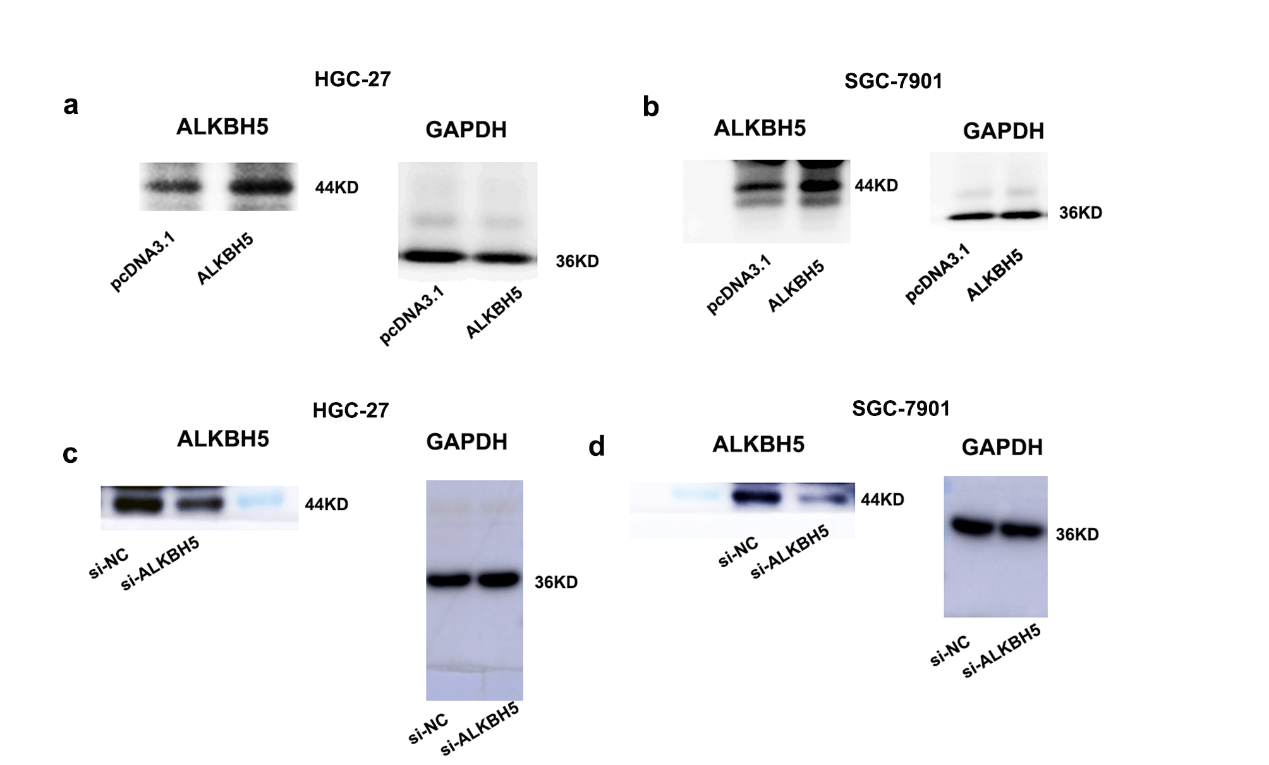


**Supplementary Fig. 4 Western blotting results of ALKBH5 expression (Fig. 6b, Fig 6d) a, b,** The relative protein expression of ALKBH5 after transfected with pcDNA3.1 or pcDNA3.1-ALKBH5 in HGC-27 and SGC-7901 cells by western blot assay. **c, d** The relative protein expression of ALKBH5 after transfected with si-NC or si-ALKBH5 in HGC-27 and SGC-7901 cells by western blot assay.

**HCG-27**


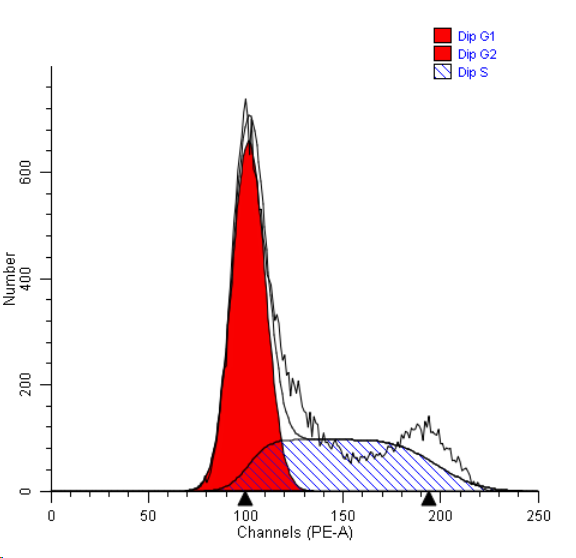

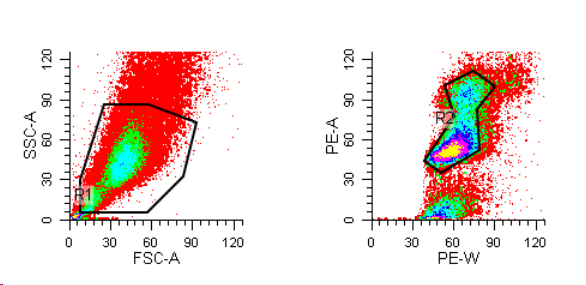


**SGC-7901**


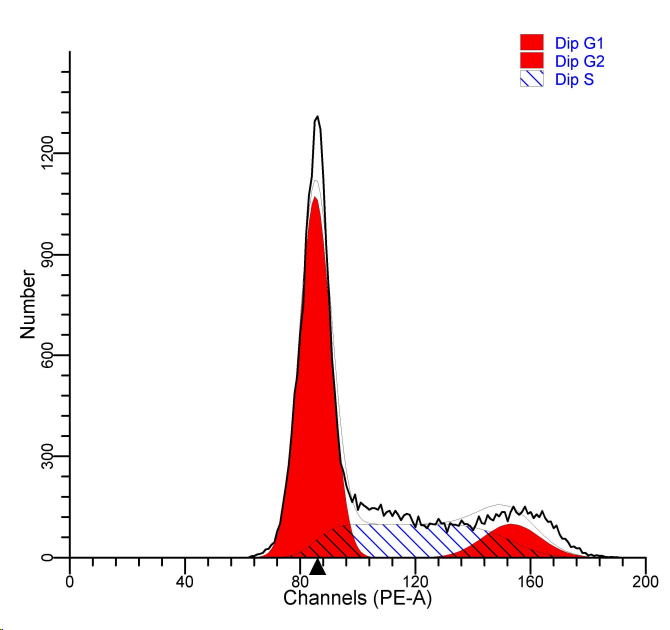

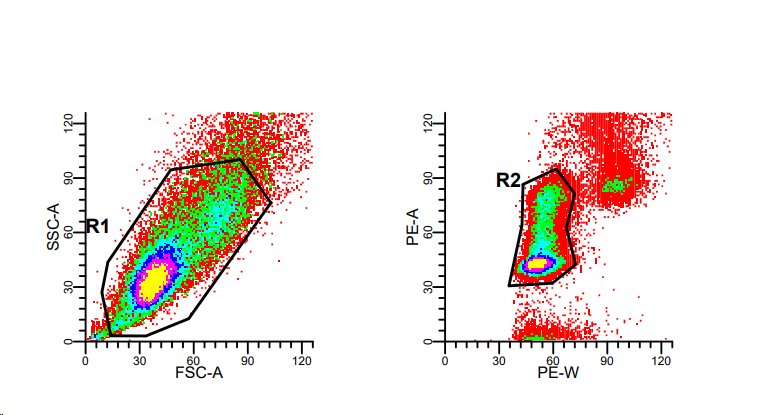


**Supplementary Fig. 5** Gating strategy for flow cytometry in analysis of cell cycle distribution for HGC-27 and SGC-7901 cells.

**Supplementary Table 1** The sequences of all primers and oligonucleotide used in the study.

| Gene | Forward primer (5’ to 3’) | Reverse primer (5’ to 3’) |
| --- | --- | --- |
| GAPDH | CGGAGTCAACGGATTTGGTCGTAT | AGCCTTCTCCATGGTGGTGAAGAC |
| U6 | CTCGCTTCGGCAGCACA | AACGCTTCACGAATTTGCGT |
| circFOXP1 | CCACATGCCTCTACCAATGGA | CAGCACTTGTTGCTGGAGGAT |
| miR-338-3p | TCCAGCATCAGTGATTTTGTTG |  |
| ALKBH5 | CGGCGA AGGCTACACTTACG | CCACCAGCTTTTGGA TCACCA |
| SOX4 | GCAAGATCATGGAGCAGTCG | GGGCCGGTACTTGTAGTCG |
| Oligonucleotide | Sense (5’ to 3’) | Antisense (5’ to 3’) |
| sh-circFOXP1-1 | CACCGAAAGGGAAAGGTTCCCGTGTCTTCAAGAGAGACACGGGAACCTTTCCCTTTTTTTTTG | GATCCAAAAAAAAAGGGAAAGGTTCCCGTGTCTCTCTTGAAGACACGGGAACCTTTCCCTTTC |
| sh-circFOXP1-2 | CACCGCTCCCAAAAGGGAAAGGTTCCTTCAAGAGAGGAACCTTTCCCTTTTGGGAGTTTTTTG | GATCCAAAAAACTCCCAAAAGGGAAAGGTTCCTCTCTTGAAGGAACCTTTCCCTTTTGGGAGC |
| miR-NC mimic | CAGUACUUUUAGUGUGUACAA |  |
| miR-338-3p mimic | UUUGAGCAGCACUCAUUUUUGC |  |
| miR-338-3p inhibitor | CAACAAAAUCACUGAUGCUGGA |  |
| miR-NC inhibitor | CAGUACUUUGUGUAGUACAA |  |
| si-NC | UUCUCCGAACGUGUCACGUTT | ACGUGACACGUUCGGAGAATT |
| si-ALKBH5 | GGAUAUGCUGCUGAUGAA ATT |  |
| si-SOX4 | GCAAACGCTGGAAGCTGCTCAAAGA |  |
