# Supplementary material for: ALKBH5-mediated m6A modification of circFOXP1 promotes gastric cancer progression by regulating SOX4 expression and sponging miR-338-3p
Source: Commun Biol. 2024 May 14;7:565. doi: 10.1038/s42003-024-06274-7 (PMC11094028; doi:10.1038/s42003-024-06274-7)
Supplement: Supplementary file 3 — Description of Additional Supplementary Files [file 42003_2024_6274_MOESM3_ESM.docx]

File name: Supplementary Data 1

Description: Source data for graphs
